# Empyema

**Published:** 1891-09

**Authors:** E. S. M’Kee

**Affiliations:** Cincinnati, Ohio


### EMPYEMA.

BY E. S. M'KEE, M. D., CINCINNATI, OHIO.

Chapin[1] calls attention to the fact that in children there is not so apt to be a bulging in the intercostal spaces on the diseased side, the lung being so soft and compressible offers the point of heart resistance to the pressure of the fluid.

Bewley[2] considers this disease due to the entrance of pus into the pleural sac, this producing micro-organisms. He thinks these organisms belong to several varieties, and that they reach the pleural sac by different routes. He describes five varieties of empyema, as follows : 1. When ordinary pyogenic micrococci made their way into the pleural sac through an opening in the chest walls, or from the lung by the bursting of a pulmonary abscess or gangrene into the pleural cavity. 2. Some cases occur in connection with croupous pneumonia, and are caused by pneumococci. 3. Some cases occur in persons afflicted with phthisis are tubercular. 4. Under various circumstances pyogenic micrococci are able to enter and live for some time in the tissues of the body without doing harm. 5. Some cases are a part of a general pyaemia.

Innermann[3] gives the following objects in the treatment of empyema : 1. Evacuate the pus already formed to prevent the reproduction of a new purulent collection ; to re-establish, as directly and as completely as possible, the normal condition of the respiratory apparatus. He considers Bulan's method of permanent aspiration to be the most rational. The method only succeeds, however, when the lung is expansible and yields to aspiration. The indication for this method is found in cases of recent empyema with pus not too thick, formed by streptococci and staphylococci, notably in bilateral empyema, where it will not do to make a double thoracic fistula.

Schede,[4] of Hamburg, does not favor repeated punctures by trocar or aspirating needle. Incision and resection is the only treatment in his opinion which is always useful and never detrimental.

Curshman[5] is well satisfied with the method of Bulan, and had sixty-three excellent results out of seventy-five cases.

Rumeberg[6] also reports very satisfactory results from this method.

Money[7] records two interesting cases of empyema complicated with pyopericardium and pulmonary abscesses; also, a case of pyohaemothorax. One was a case of pneumonia complicating parturition, and resulting in multiple abscesses of the lung.

The ætiology of empyema in children is very carefully studied by Koplick.[8] In his investigations he adhered closely to the method employed by the Koch school. Careful bacteriological investigations were made in twelve cases of empyema, the cases being divided into five groups. 1. Those in which the bacteriscopic results are not uniform, and the micro-organisms found not diagnostic. 2. Those in which he was not able to establish the presence of the pneumococcus of Fraenkel and Weichelbaum in the purulent exudate. 3. Empyema occuring in tubercular subjects. 4. Those cases in which a focus of suppuration situated outside the chest can be pointed to with a degree of probability as the possible source of infection. His investigations led him to the conclusion that a large proportion of empyemas in children follow or complicate processes in the lung of an acute character. With early and efficient treatment, these cases can be met cheerfully. Even those whose ætiology is uncertain, do not hold out such a bad prognosis to the patient. The tubercular and the pyemic cases are the stumbling blocks to pediatric practice.

A case of encysted empyema in which the value of using the exploring needle is reported by Davidson.[9]

In this case all pain was referred to the right lumbar region. Bacteriological investigations have been made by

Shaw. In four cases where the pus was examined, the pneumococcus was found in each. The pus from two of these was inoculated into a rabbit and a mouse with the result that the pneumococcus was obtained in a pure culture. The micro-organism in the pus of all examined was in a form never before seen, viz.: in chains of from 4-7 in number, and surrounded by a continuous capsule. Some of these had peculiar hooked shapes.

The editor of the *Journal of the American Medical Association* cites the wonderful increase during the past year of empyemas, the influenza being the primary disease in many cases. He states the origin of these empyemas as: General infection of the blood with influenza and consequent saturation of its germicidal quality; invasion of portions of the lung with pneumonia producing parasites, and consequent interruption of the lymph currents from the pulmonary pleura over the affected area, resulting in serous pleuritic effusion; invasion of the lymph spaces in the pneumonic area by pus-producing bacteria which advance from the larger to the smaller bronchioles and to the lymphatics. These are then carried in the lymph in its abnormal course into the pleural cavity; here the bacteria multiply in the effused serum until the pleural surfaces are so modified by the products of bacterial growth that absorption is arrested and these surfaces become one continuous pyogenic membarne.

The pathology of empyema is the subject of an able paper by Bewley.[10] He concludes that empyema is always caused by micro-organisms, but that they are of different species, not of one specific variety. Occasionally ordinary pus-producing and putrefactive bacteria get into the pleural cavity through some opening into the chest wall or lung. Some cases are associated with croupous pneumonia and are caused by the pneumococcus. Some are due to the action of the tubercle bacillus; others are pyaemic. In some cases pus-producing micrcocci, which have in some way found entrance to the blood, but unassisted are not able to develop in the body, find a suitable locality for development in an inflamed pleura or serous effusion, and under their influence a serous effusion becomes purulent. The incision into the center of the empyaemic cavity at the upper border of a rib, the washing out of the pus once or twice a day with the most scrupulous antiseptic precautions, is a method which, in the hand of Quine[11] and others, has proven very valuable. When the discharge becomes serous, after a week or ten days, the antiseptic irrigations are redoubled in amount and care for a few days. The incision must be closed, so that all ingress of air is prevented by the coaptation of the lips of the incision, while the air within the cavity is absorbed or forced out of the incision by coughing.

1. Archives of Pedriatrics, June, 1890.
2. Dublin Journal Med. Sc., August, 1890.
3. Boston Med. and Surg. Journal, May 15, 1890. Tr. 9th Congress of International Medicine, Vienna, 1890. LaSemaine Medicale.
4. Loc cit.
5. Loc cit.
6. Berlin Klin. Wochenschrift, 2 June, 1890.
7. Lancet, Oct. 18, 1890. Also, Vol. I, 1889.
8. Australian Medical Gazette, April, 1890.
9. Journal American Medical Association, Sept 13, 1890.
10. Archives of Pedriatrics, Oct., 1890.
11. Dublin Journal of the Medical Sciences, Nov. 1, 1890.

